# Antimicrobial resistance in *Enterococcus* isolated from western Canadian cow-calf herds

**DOI:** 10.1186/s12917-023-03843-6

**Published:** 2024-01-03

**Authors:** Jayce D. Fossen, John R. Campbell, Sheryl P. Gow, Nathan Erickson, Cheryl L. Waldner

**Affiliations:** 1 Large Animal Clinical Sciences, Western College of Veterinary Medicine, 52 Campus Dr, Saskatoon, Saskatchewan S7N 5B4 Canada; 2 Public Health Agency of Canada, Saskatoon, Saskatchewan S7N 5B4 Canada

**Keywords:** Antimicrobial resistance, Cow-calf herds, Enterococcus

## Abstract

**Background:**

Data on antimicrobial resistance (AMR) in cow-calf herds is limited and there have been no Canadian studies examining AMR in *Enterococcus* in cow-calf herds. *Enterococcus* is a ubiquitous Gram-positive indicator of AMR for enteric organisms that is also important in human health. The objective of this study was to describe AMR in specific *Enterococcus* species of interest from cow-calf herds; highlighting differences in AMR among isolates from cows and calves and samples collected in the spring and fall. Isolates (*n* = 1505) were examined from 349 calves and 385 cows from 39 herds in the spring of 2021 and 413 calves from 39 herds and 358 cows from 36 herds in the fall of 2021. *Enterococcus* species were identified using Matrix-Assisted Laser Desorption Ionization Time-Of-Flight mass spectrometry (MALDI-TOF MS) and antimicrobial susceptibility testing was completed based on a prioritization scheme for importance to human health and using the National Antimicrobial Resistance Monitoring System (NARMS) Gram positive Sensititre broth microdilution panel.

**Results:**

Resistance was observed to at least one antimicrobial in 86% (630/734) of isolates from the spring and 84% (644/771) of isolates from the fall. The most common types of resistance across all species were: lincomycin, quinupristin/dalfopristin, daptomycin, ciprofloxacin, and tetracycline. However, the proportion of isolates with AMR varied substantially based on species. Multiclass resistance, defined as resistance to ≥3 antimicrobial classes after excluding intrinsic resistance, was highest in isolates from calves in the spring (6.9%) (24/349) and cows in the fall (6.7%) (24/357). Differences in resistance were seen between cows and calves in the spring and fall as well as across seasons, with no differences seen between cows and calves in the fall.

**Conclusions:**

While most *Enterococcus* isolates were resistant to at least one antimicrobial, questions remain regarding species differences in intrinsic resistance and the accuracy of certain antimicrobial breakpoints for specific *Enterococcus* spp. As a result, some species-specific AMR profiles should be interpreted with caution. Despite these constraints, *Enterococcus* species are important indicator organisms for AMR and resulting data can be used to inform stewardship initiatives.

**Supplementary Information:**

The online version contains supplementary material available at 10.1186/s12917-023-03843-6.

## Background

Enterococci have been the focus of a number of recent studies across the One Health continuum and act as an important indicator species for monitoring AMR in Gram-positive organisms [1–3]. *Enterococcus* is also of interest because of its capacity to transfer AMR genes among species within the genus and to other pathogens through mobile genetic elements such as plasmids and transposons [4, 5]. *Enterococcus* has been traditionally reported in public health as a sentinel for fecal contamination [6]. However, because these organisms can readily acquire antimicrobial resistance genes, their adaptability to numerous host species and environments, and their importance as opportunistic human pathogens, *Enterococcus* spp. are increasingly being monitored as part of national AMR surveillance programs [7–10].

Antimicrobial resistance in *Enterococcus* spp. is of particular interest in beef cattle because of the importance of macrolides in both managing bovine respiratory disease (BRD) and in preventing liver abscesses [11]. Macrolide resistance in *Enterococcus* spp. from beef cattle has been associated with macrolide use either as injectable products to manage BRD or in feed for liver abscesses [11, 12]. Macrolide resistance in enterococci is an important concern in human medicine because of the similarity in location and potential for co-selection between macrolide and vancomycin resistance genes [11]. The threat from vancomycin resistant enterococci (VRE) is increasing [13] and VRE are considered among the most serious nosocomial pathogens [14, 15].

Previous work has described differences between *Enterococcus* spp. isolated from feedlots and sources more likely to be of human origin [1]. However, this research has not been repeated in cow-calf herds where both antimicrobial use (AMU) and animal management differ greatly from large North American feedlots [16, 17]. Certain species of *Enterococcus*, specifically *E. faecalis* and *E. faecium*, are common causes of *Enterococcus*-related diseases in humans and resistant enterococcal infections have become an increasing threat to human health [1, 13]. *E. faecalis* and *E. faecium* are also found in cattle [1, 8]. While, there have been some studies that described AMR for *Enterococcus* spp. isolated from beef cattle [1, 2, 7, 9, 11, 12, 18], beef processing plants and ground beef products [19] in Canada, there were no identified publications investigating AMR for *Enterococcus* spp. in Canadian cow-calf herds [3]. Antimicrobial resistance data for *Enterococcus* spp. has previously been reported for cow-calf herds in the United States from 2007 to 08 and 2017, and more recently from beef herds in California [8, 10, 20]. Finally, one Australian study examined resistance in *Enterococcus* recovered from cattle at the time of arrival in the feedlot [21].

Although the cow-calf industry is a key component to Canadian agriculture and represents the most numerous livestock operation type in Canada with 54,000 herds across the country [22], this sector is not currently part of federal AMR surveillance initiatives. Historic baseline data primarily focused on *Escherichia coli (E. coli)* [23–25]. *E. coli* is also a commonly reported sentinel species for monitoring AMR for enteric organisms; however, this one organism does not provide meaningful AMR data for all antimicrobials important to veterinary and human health. For example, *E. coli* is intrinsically resistant to many macrolides used in veterinary medicine due to the impermeable nature of the Gram-negative outer membrane to many of these compounds [26]. Therefore, describing AMR in *Enterococcus* bacteria in the cow-calf sector will allow for a deeper understanding of potential relationships among AMR in animal agriculture, the environment, and human health.

The objective of this observational study was to describe AMR in *Enterococcus* spp. that are of interest to human health, and where those species were not identified, then for the most identified *Enterococcus* spp. from beef herds in western Canada. The second objective was to compare the frequency of AMR in species of interest between cows and calves in the spring and the fall. Based on previous studies of *E. coli* in cow-calf herds [23, 24], the study authors hypothesized that the prevalence of AMR would differ between cows and calves within season, and between seasons for cows and for calves.

## Results

### Study population and recovery of *Enterococcus* spp.

Of the 50 herds initially enrolled, 39 herds provided fecal samples in both the spring and fall. Of those herds, the largest proportion came from Alberta (51%, 20/39), followed by Saskatchewan (23%, 9/39), Manitoba (13%, 5/39), and British Columbia (13%, 5/39).

In the spring of 2021, *Enterococcus* spp. were isolated from 89% (349) of the 390 sampled calves and 99% (385) of the 387 cows. Participating herds started calving between December 2020, and May of 2021. Most herds started to calve in March (33%, 13/39) and April (28%, 11/39). Spring samples were collected following the peak of calving in March through August, with most samples (59%, 23/39) collected in June. The average age of calves for which samples were collected in the spring was 8 weeks (SD, 5 weeks). All herds collected samples from cows and calves at the same time.

In the fall of 2021, 419 calves and 359 cows were sampled, with isolation rates of 99% (413/419) and 99% (358/359). Additional calf samples were provided by three herds in the fall of 2021 instead of providing fecal samples from cows. The fall samples were collected in September though January, with most collected in October (33%, 13/39) and November (44%, 17/39). Calf age at time of fall sample collection was not available. Most herds collected fall samples from cows and calves at the same time. Four of 36 herds collected samples from cows and calves on different dates; three herds collected both samples from both groups within the same week, and the final herd collected samples from cows in October, and calves in January. Antimicrobial use histories were not available at the time of convenience sampling for individual animals; however, no animals were reported to be sick and requiring antimicrobial treatment at the time of sample collection.

### Summary of recovered *Enterococcus* isolates

Based on the priority selection protocol for this study (*Enterococcus faecalis*, *Enterococcus faecium*, *Enterococcus hirae*, then most common other), the isolates most frequently selected for further AST testing in the spring from calves were *E. faecalis* (24%, 85/349) (Table 1). Because no *E. faecium*, *E. faecalis*, or *E. hirae* were identified in many of the cows, *Enterococcus casseliflavus* was the most common species identified in cows in the spring (Table 1). The primary species selected from both calves and cows in the fall was also *E. casseliflavus* (Table 2). *E. casseliflavus* was second most likely to be recovered for further testing from calves in the spring (Table 1). *E. hirae* was the second most common *Enterococcus* spp. recovered in three of the four groups. *E. hirae* was recovered from cows in the spring and fall, as well as calves in the fall (Tables 1a, b).

**Table 1:** Prevalence of antimicrobial resistance (%) for selected *Enterococcus* isolates recovered in the spring of 2021 from 390 calves and 387 cows from 39 herds

Relative frequency of isolates	Enterococcus isolates from calves (*n* = 349) in the spring					Enterococcus isolates from cows (*n* = 385) in the spring				
	*E. faecalis*	*E. casseliflavus*	*E. hirae*	*E. faecium*	*E. species* ^*a*^	*E. casseliflavus*	*E. hirae*	*E. faecalis*	*E. faecium*	*E. species*^*b*^
	24% (85/349)	23% (79/349)	21% (75/349)	14% (50/349)	17% (60/349)	42% (162/385)	18% (70/385)	8.8% (34/385)	7.0% (27/358)	24% (92/385)
Antimicrobials tested:	Percentage of isolates resistant to tested antimicrobials (Number resistant/Number isolates tested)									
	**Category I: Very High Importance** ^c^									
Ciprofloxacin	2.4% (2/85)	3.8% (3/79)	1.3% (1/75)	60% (30/50)	3.3% (2/60)	3.1% (5/162)	1.4% (1/70)	0% (0/34)	48% (13/27)	1.1% (1/92)
Daptomycin^d^	2.4% (2/85)	1.3% (1/79)	64% (48/75)	32% (16/50)	10% (6/60)	0% (0/162)	56% (39/70)	0% (0/34)	33% (9/27)	5.4% (5/92)
Linezolid	2.4% (2/85)	0% (0/79)	0% (0/75)	0% (0/50)	0% (0/60)	0% (0/162)	0% (0/70)	2.9% (1/34)	0% (0/27)	0% (0/92)
Tigecycline ^e^	1.2% (1/85)	0% (0/79)	1.3% (1/75)	10% (5/50)	1.7% (1/60)	1.9% (3/162)	0% (0/70)	0% (0/34)	7.4% (2/27)	0% (0/92)
Vancomycin ^f^	0% (0/85)	*0% (0/79)*	0% (0/75)	0% (0/50)	0% (0/60)	*0% (0/162)*	0% (0/70)	0% (0/34)	0% (0/27)	0% (0/92)
	**Category II: High Importance** ^c^									
Erythromycin	8.2% (7/85)	2.5% (2/79)	2.7% (2/75)	8% (4/50)	0% (0/60)	3.7% (6/162)	4.3% (3/70)	2.9% (1/34)	0% (0/27)	0% (0/92)
Gentamicin ^f^	*0% (0/85)*	*0% (0/79)*	0% (0/75)	*0% (0/50)*	0% (0/60)	*0% (0/162)*	0% (0/70)	*0% (0/34)*	*0% (0/27)*	0% (0/92)
Kanamycin ^f^	*5.9% (5/85)*	*0% (0/79)*	1.3% (1/75)	*6% (3/50)*	0% (0/60)	*1.2% (2/162)*	0% (0/70)	*0% (0/34)*	*3.7% (1/27)*	0% (0/92)
Lincomycin ^f^	*NA*	*100% (79/79)*	*73% (55/75)*	*58% (29/50)*	*92% (55/60)*	*98% (158/162)*	*84% (59/70)*	*NA*	*48% (13/27)*	*91% (84/92)*
Penicillin	0% (0/85)	0% (0/79)	0% (0/75)	0% (0/50)	0% (0/60)	0% (0/162)	0% (0/70)	0% (0/34)	3.7% (1/27)	0% (0/92)
Quinupristin/ Dalfopristin ^f^	*NA*	*41% (32/79)*	4% (3/75)	14% (7/50)	6.7% (4/60)	*38% (62/162)*	5.7% (4/70)	*NA*	3.7% (1/27)	*2.2% (2/92)*
Streptomycin ^f^	*8.2% (7/85)*	*1.3% (1/79)*	4% (3/75)	*8% (4/50)*	0% (0/60)	*1.9% (3/162)*	0% (0/70)	*2.9% (1/34)*	*0% (0/27)*	1.1% (1/92)
Tylosin	8.2% (7/85)	2.5% (2/79)	2.7% (2/75)	8% (4/50)	0% (0/60)	4.3% (7/162)	4.3% (3/70)	2.9% (1/34)	0% (0/27)	1.1% (1/92)
	**Category III: Medium Importance** ^c^									
Chloramphenicol	0% (0/75)	0% (0/79)	0% (0/75)	0% (0/50)	0% (0/60)	0% (0/162)	0% (0/70)	5.9% (2/34)	0% (0/27)	0% (0/92)
Nitrofurantoin	0% (0/85)	0% (0/79)	0% (0/75)	12% (6/50)	6.7% (4/60)	0% (0/162)	0% (0/70)	0% (0/34)	11% (3/27)	5.4% (5/92)
Tetracycline	40% (34/85)	5.1% (4/79)	5.3% (4/75)	18% (9/50)	5% (3/60)	1.9% (3/162)	5.7% (4/70)	5.9% (2/34)	7.4% (2/27)	4.3% (4/92)

^a, b^: *E. durans, E. gallinarum, E. mundtii, E. species;* ^c^ Government of Canada 2009 [27] ^d^ Daptomycin breakpoints for susceptible vary for *E. faecium* as compared to other species [28–31]; ^e^ No CLSI breakpoint for resistance for tigecycline, rather only for susceptible; the numbers reflect isolates that are not susceptible vs resistant [32].; ^f^ *Intrinsic resistance*: aminoglycosides (gentamicin, kanamycin, streptomycin), (*E.* spp) [33], lincosamides, (*E. faecalis*) [14, 33, 34], (*E. casseliflavus*) [34]; quinupristin/dalfopristin (*E. faecalis*) [14, 33, 34], (*E. casseliflavus*) [33, 34]; vancomycin (*E. casseliflavus*) [33]; *NA – E. faecalis resistance to either lincomycin or quinupristin/dalfopristin were not included in any analysis due to intrinsic resistance due to the presence of the lsa gene* [14]

**Table 2:** Prevalence of antimicrobial resistance (%) for selected *Enterococcus* isolates recovered in the fall of 2021 from 419 calves from 39 herds and 359 cows from 36 herds

Relative frequency of isolates	Enterococcus isolates from calves (*n* = 413) in the fall					Enterococcus isolates from cows (*n* = 358) in the fall				
	*E. casseliflavus*	*E. hirae*	*E. faecalis*	*E. faecium*	*E. species*^*a*^	*E. casseliflavus*	*E. hirae*	*E. faecalis*	*E. faecium*	*E. species*^*b*^
	36% (149/413)	22% (89/413)	15% (62/413)	11% (45/413)	16% (68/413)	37% (131/358)	16% (56/358)	13% (48/358)	12% (43/358)	22% (80/358)
Antimicrobials tested:	Percentage of isolates resistant to tested antimicrobials (Number resistant/Number isolates tested)									
	**Category I: Very High Importance** ^c^									
Ciprofloxacin	14% (21/149)	1.1% (1/89)	0% (0/62)	64% (29/45)	0% (0/68)	15% (20/131)	1.8% (1/56)	2.1% (1/48)	65% (28/43)	3.8% (3/80)
Daptomycin ^d^	0% (0/149)	45% (40/89)	1.6% (1/62)	13% (6/45)	12% (8/68)	0.8% (1/131)	50% (28/56)	1.8% (1/48)	12% (5/43)	10% (8/80)
Linezolid	0% (0/149)	0% (0/89)	0% (0/62)	0% (0/45)	0% (0/68)	0% (0/131)	0% (0/56)	0% (0/48)	0% (0/43)	0% (0/80)
Tigecycline ^e^	1.3% (2/149)	0% (0/89)	0% (0/62)	6.7% (3/45)	1.5% (1/68)	1.5% (2/131)	1.8% (1/56)	4.3% (2/48)	4.7% (2/43)	0% (0/80)
Vancomycin^f^	*0% (0/149)*	0% (0/89)	0% (0/62)	0% (0/45)	0% (0/68)	*0% (0/131)*	0% (0/56)	0% (0/48)	0% (0/43)	0% (0/80)
	**Category II: High Importance** ^c^									
Erythromycin	2.7% (4/149)	2.2% (2/89)	4.8% (3/62)	4.4% (2/45)	0% (0/68)	2.3% (3/131)	0% (0/56)	2.1% (1/48)	2.3% (1/43)	0% (0/80)
Gentamicin^f^	*0% (0/149)*	0% (0/89)	*0% (0/62)*	*0% (0/45)*	0% (0/68)	*0% (0/131)*	0% (0/56)	*0% (0/48)*	*0% (0/43)*	0% (0/80)
Kanamycin^f^	*0% (0/149)*	1.1% (1/89)	*3.2% (2/62)*	*0% (0/45)*	0% (0/68)	*0% (0/131)*	0% (0/56)	*0% (0/48)*	*0% (0/43)*	0% (0/80)
Lincomycin^f^	*99%(148/149)*	*67% (60/89)*	*NA*	*33% (15/45)*	*91% (62/68)*	*99% (130/131)*	*77% (43/56)*	*NA*	*33% (14/43)*	*98% (78/80)*
Penicillin	0% (0/149)	0% (0/89)	0% (0/62)	0% (0/45)	0% (0/68)	0% (0/131)	0% (0/56)	0% (0/48)	0% (0/43)	0% (0/80)
Quinupristin/ dalfopristin^f^	*34% (50/149)*	2.2% (2/89)	*NA*	6.7% (3/45)	1.5% (1/68)	*32% (42/131)*	5.4% (3/56)	*NA*	19% (8/43)	7.5% (6/80)
Streptomycin^f^	*0% (0/149)*	5.6% (5/89)	*6.5% (4/62)*	*0% (0/45)*	0% (0/68)	*0% (0/131)*	1.8% (1/56)	*0% (0/48)*	*0% (0/43)*	6.3% (5/80)
Tylosin	3.4% (5/149)	2.2% (2/89)	6.5% (4/62)	0% (0/45)	1.5% (1/68)	3.1% (4/131)	0% (0/56)	0% (0/48)	0% (0/43)	0% (0/80)
	**Category III: Medium Importance** ^c^									
Chloramphenicol	0.7% (1/149)	0% (0/89)	3.2% (2/62)	0% (0/45)	0% (0/68)	0% (0/131)	0% (0/56)	0% (0/48)	0% (0/43)	0% (0/80)
Nitrofurantoin	0% (0/149)	0% (0/89)	0% (0/62)	42% (19/45)	12% (8/68)	0% (0/131)	1.8% (1/56)	0% (0/48)	25% (11/43)	5.0% (4/80)
Tetracycline	0% (0/149)	20% (18/89)	15% (9/62)	6.7% (3/45)	8.8% (6/68)	3.8% (5/131)	14% (8/56)	11% (5/48)	12% (5/43)	6.3% (5/80)

^a, b^: *E. durans, E. gallinarum, E. mundtii, E. species;* ^c^ Government of Canada 2009 [27] ^d^ Daptomycin breakpoints for susceptible vary for *E. faecium* as compared to other species [28–31] ^e^ No CLSI breakpoint for resistance for tigecycline, rather only for susceptible; the numbers reflect isolates that are not susceptible vs resistant [32].;^f^ *Intrinsic resistance*: aminoglycosides (gentamicin, kanamycin, streptomycin), (*E.* spp) [33], lincosamides, (*E. faecalis*) [14, 34], (*E. casseliflavus*) [34]; quinupristin/dalfopristin (*E. faecalis*) [14, 33, 34], (*E. casseliflavus* )[33, 34]; vancomycin (*E. casseliflavus*) [33]; *NA – E. faecalis resistance to either lincomycin or quinupristin/dalfopristin were not included in any analysis due to intrinsic resistance due to the presence of the lsa gene* [14]

*E. faecalis* was nearly twice as likely to be recovered from calves in the spring compared to calves in the fall (Table 3). *E. faecalis* and *E. faecium* were also 3.6 and 2.4 times more likely to be reported in calves in the spring compared to cows in the spring (Table 3). *E. hirae* was more likely to be recovered from calves in the fall than cows in the fall. Whereas *E. faecium* was more likely to be recovered from cows in the fall compared to cows in the spring (Table 3).

**Table 3:** Conditional associations describing differences in the occurrence of *Enterococcus* isolates recovered in the spring of 2021 from 390 calves and 387 cows from 39 herds and in the fall of 2021 from 419 calves from 39 herds and 359 cows from 36 herds

	Fall Calf vs. Spring Calf (ref)		Spring Cow vs. Spring Calf (ref)		Fall Cow vs. Fall Calf (ref)		Fall Cow vs. Spring Cow (ref)		
	Odds Ratio (95% CI)	*P*-value	Odds Ratio (95% CI)	*P*-value	Odds Ratio (95% CI)	*P*-value	Odds Ratio (95% CI)	*P*-value	ICC^b^
*E. faecalis*^*a*^	0.52 (0.36-0.77)	0.001	0.28 (0.18-0.44)	< 0.001	0.83 (0.55-1.28)	0.41	1.57 (0.97-2.53)	0.07	0.13
*E. faecium*	0.67 (0.43-1.05)	0.08	0.42 (0.26-0.70)	0.001	1.15 (0.73-1.83)	0.53	1.83 (1.10-3.08)	0.02	0.13
*E. hirae*	0.98 (0.68-1.40)	0.91	0.78 (0.54-1.14)	0.20	0.69 (0.46-1.00)	0.05	0.85 (0.57-1.27)	0.43	0.11
*E. casseliflavus*	2.19 (1.56-3.09)	< 0.001	2.88 (2.05-4.07)	< 0.001	1.01 (0.74-1.39)	0.93	0.77 (0.56-1.06)	0.11	0.18
*E. species*	0.98 (0.66-1.46)	0.94	1.55 (1.06-2.25)	0.02	1.42 (0.97-2.07)	0.07	0.90 (0.63-1.29)	0.57	0.09

ref – reference group for interpretation of odds ratio.

^a^Species are rank ordered by selection preference criteria

^b^*ICC* Intraclass Correlation Coefficient

### Antimicrobial resistance for *Enterococcus* isolates

The most common types of resistance across all species were: lincomycin, quinupristin/dalfopristin, daptomycin, ciprofloxacin, and tetracycline (Table B1) even when *E. faecalis* was excluded from the summary due to intrinsic resistance. Most isolates from both calves (82%, 287/349) and cows (89%, 343/385) in the spring, as well as calves (83%, 342/413) and cows 85% (302/358) in the fall, were resistant to at least one of the tested antimicrobials.

### Spring calves

In *E. casseliflavus* isolates recovered from calves in the spring, resistance to lincomycin followed by quinupristin/dalfopristin were the most common (Table 1). For *E. hirae* isolates, the most common resistance was to lincomycin with daptomycin resistance being the next most common resistance detected (Table 1) In *E. faecium*, ciprofloxacin resistance was the most common followed by resistance to lincomycin (Table 1). Tetracycline resistance was the most common resistance detected for *E. faecalis* (Table 1).

### Spring cows

For cows in the spring similar resistance patterns to spring calves were observed for the four primary target species. For *E. casseliflavus* lincomycin and quinupristin/dalfopristin were the first and second most common resistance detected (Table 1). For *E. hirae* isolates from cows in the spring, lincomycin resistance was the most common followed by daptomycin (Table 1). *Enterococcus faecium* isolates recovered from cows in the spring were most likely to be resistant to ciprofloxacin and lincomycin, while daptomycin was the next most common resistance detected (Table 1). For *E. faecalis* recovered from cows in the spring, tetracycline and chloramphenicol were the most common resistances (Table 1).

### Fall calves

For calves in the fall, *E. casseliflavus* were most likely to be resistant to lincomycin followed by quinupristin/dalfopristin (Table 2). Lincomycin was also the most common resistance for *E. hirae* with daptomycin the second most common (Table 2). As with the spring, the *E. faecium* isolates recovered from calves in the fall showed the greatest resistance to ciprofloxacin (Table 2). The second most frequently observed resistance for *E. faecium* isolates recovered from calves in the fall was to nitrofurantoin (Table 1). Tetracycline was the most common resistance seen in *E. faecalis* (Table 2).

### Fall cows

Isolates recovered from cows in the fall had similar AMR profiles to those recovered from calves in the fall. Lincomycin and quinupristin/dalfopristin were the first and second most common resistance detected in *E. casseliflavus* (Table 2). Lincomycin resistance in *Enterococcus hirae* isolates from cows in the fall were most common followed by resistance to daptomycin (Table 2). *Enterococcus faecium* were most likely to be resistant to ciprofloxacin with *E. faecalis* isolates recovered from cows in the fall most commonly resistant to tetracycline (Table 2). The second most common resistance in *E. faecium* isolates was to lincomycin, while the second most common resistance for *E. faecalis* was tigecycline (Table 2).

### Multiclass resistance and resistance to antimicrobials of very high importance to human health

The frequency of muti-class resistant (≥3 classes) enterococci bacteria and isolates resistant to > 1 antimicrobial was very similar for calves in the spring and cows in the fall (Table 4). One calf isolate from the spring was resistant to six classes of antimicrobials (Table 4), including fluoroquinolones, glycylcyclines, lincosamides, lipopeptides, macrolides, and tetracyclines. Additionally, one isolate from cows in the spring was resistant to six classes of antimicrobials (Table 4), including lincosamides, lipopeptides, macrolides, oxazolidinones penicillins, phenicols, and streptogramins.

**Table 4:** Antimicrobial class resistance % (n) for all *Enterococcus* spp.^a,b^ isolates recovered in 2021

Resistant to	Spring Calf (*n* = 349)	Spring Cow (*n* = 385)	Fall Calf (*n* = 413)	Fall Cow (*n* = 358)
Pan-susceptible	17% (61)	10% (40)	17% (71)	15% (55)
1 class	52% (180)	68% (262)	52% (216)	57% (202)
> 1 Class	31% (108)	21% (82)	30% (124)	28% (100)
2 classes	24% (84)	17% (66)	26% (106)	21% (76)
3 classes	3.7% (13)	3.1% (12)	3.6% (15)	5.1% (19)
4 classes	1.7% (6)	0.8% (2)	0.7% (3)	1.4% (5)
5 classes	1.1% (4)	0.3% (1)	0% (0)	0% (0)
6 classes	0.3% (1)	0.3% (1)	0% (0)	0% (0)
Multiclass resistance ≥3 classes all species^b^	6.9% (24)	4.2% (16)	4.4% (18)	6.7% (24)
*• E. casseliflavus*	0% (0/79)	0.6% (1/162)	1.3% (2/149)	3.8% (5/131)
*• E. faecalis*	2.4% (2/85)	2.9% (1/34)	3.2% (2/62)	0% (0/48)
*• E. faecium*	30% (15/50)	18.5% (5/27)	20% (9/45)	20.9% (9/43)
*• E. hirae*	6.7% (5/75)	7.1% (5/70)	5.6% (5/89)	5.4% (3/56)
*• E. species*	3.3% (2/60)	4.3% (4/92)	0% (0/68)	8.8% (7/80)
Resistance to a Category I antimicrobial^c^	35% (121)	21% (79)	27% (112)	29% (103)

^a^Resistance of *E. faecalis* to quinupristin/dalfopristin and lincomycin are not included because of intrinsic resistance [14]

^b^Intrinsic resistance not included in determination of multiclass resistance [33]

^c^Government of Canada 2009 [27]

For calves in the spring, there were 19 unique antimicrobial class combinations for which multiclass resistant isolates were observed, while 14 multiclass combinations were found in resistant isolates from cows. The most common multiclass resistance combination from calves (17%, 4/24) consisted of fluoroquinolones, lincosamides, and lipopeptides,. While in cows in the spring, there were two equally common multiclass resistant combinations (13%, 2/16) were lincosamides, lipopeptides, and streptogramins as well as lincosamides, lipopeptides, and tetracyclines.

In calves in the fall, the most common multiclass resistant combination was similar to one of the patterns seen in cows in the spring. Seventeen percent (3/18) of multiclass resistant isolates were resistant to the antimicrobial classes of lincosamides, lipopeptides, and tetracyclines. As with cows in the spring, there were two multiclass resistance patterns observed equally in prominence in cows in the fall. These combinations were each observed in 13% (3/24) of multiclass resistant isolates. The combinations included fluoroquinolones, lincosamides, and lipopeptides, as well as lincosamides, streptogramins, and tetracyclines.

Resistance to at least one Category I antimicrobial was most common in isolates from calves in the spring (Table 4).

### Difference in AMR between calves and cows and between isolates from spring and fall

Significant differences in *Enterococcus* resistance profiles were observed between animal classes within seasons and within animal classes between seasons (Table B2). The exception to this was the fall, where no significant differences were measured in the species-specific resistance between cows and calves within the season (Table 5). Differences in resistant profiles were found in every species tested, with *E. faecium* being the species for which the largest number of significant differences were observed.

**Table 5:** Summary of differences in the odds of antimicrobial resistance for individual *Enterococcus* species isolated from calves and cows in the spring and in the fall accounting for clustering within herds using generalized estimation equations

		Fall Calf vs. Spring Calf (ref)		Spring Cow vs. Spring Calf (ref)		Fall Cow vs. Fall Calf (ref)		Fall Cow vs. Spring Cow (ref)		
Species	Antimicrobial	Odds Ratio (95% CI)	*P*-value	Odds Ratio (95% CI)	*P*-value	Odds Ratio (95% CI)	*P*-value	Odds Ratio (95% CI)	*P*-value	ICC
*E. faecalis*^*a*^	Streptomycin	0.61 (0.12-3.14)	0.55	0.34 (0.02-5.66)	0.46	Not Estimable	> 0.99	Not Estimable	> 0.99	0.61
*E. faecalis*	Tetracycline	**0.21 (0.08-0.54)**	**0.001**	**0.09 (0.02-0.43)**	**< 0.01**	0.27 (0.05-1.39)	0.12	0.64 (0.08-5.17)	0.68	0.16
*E. faecalis*	Tylosin	0.74 (0.18-3.05)	0.68	0.37 (0.04-3.56)	0.39	Not Estimable	> 0.99	Not Estimable	> 0.99	0.28
*E. faecalis*	Pan-susceptible	**4.36 (1.72-11.1)**	**< 0.01**	**8.15 (2.17-30.7)**	**< 0.01**	2.51 (0.68-9.22)	0.17	1.34 (0.27-6.72)	0.72	0.28
*E. faecalis*	> 1 Antimicrobial Class	0.61 (0.15-2.54)	0.50	0.25 (0.02-2.51)	0.24	Not Estimable	> 0.99	Not Estimable	> 0.99	0.33
*E. faecalis*	Multiclass resistant	1.46 (0.17-12.2)	0.73	1.18 (0.09-15.5)	0.90	Not Estimable	> 0.99	Not Estimable	> 0.99	0.25
*E. faecium*	Ciprofloxacin	1.36 (0.53-3.55)	0.52	0.56 (0.19-1.66)	0.29	0.97 (0.09-0.35)	0.96	2.60 (0.73-7.93)	0.15	0.20
*E. faecium*	Nitrofurantoin	**8.53 (2.24-32.5)**	**< 0.01**	0.81 (0.16-4.19)	0.80	0.31 (0.09-1.09)	0.07	Not Estimable	> 0.99	0.33
*E. faecium*	Quinupristin/dalfopristin	0.40 (0.09-1.84)	0.24	0.23 (0.02-2.1)	0.20	3.35 (0.75-15.0)	0.11	5.90 (0.63-55.8)	0.12	0.17
*E. faecium*	Tetracycline	0.30 (0.06-1.42)	0.13	0.25 (0.04-1.54)	0.14	2.68 (0.49-14.8)	0.26	3.20 (0.39-26.6)	0.28	0.32
*E. faecium*	Tigecycline	0.34 (0.04-2.85)	0.32	0.49 (0.07-3.68)	0.50	0.91 (0.1-8.61)	0.94	0.64 (0.05-8.19)	0.73	0.46
*E. faecium*	Pan-susceptible	1.53 (0.32-7.23)	0.59	1.25 (0.19-8.00)	0.81	1.05 (0.25-4.50)	0.95	1.28 (0.22-7.53)	0.78	0
*E. faecium*	> 1 Antimicrobial Class	0.93 (0.38-2.29)	0.88	0.48 (0.17-1.33)	0.16	0.70 (0.27-1.79)	0.45	1.37 (0.46-4.04)	0.57	0.11
*E. faecium*	Multiclass resistant	0.59 (0.22-1.63)	0.31	0.50 (0.15-1.68)	0.26	1.11 (0.37-3.35)	0.85	1.32 (0.34-5.07)	0.69	0.08
*E. hirae*	Tetracycline	**5.53 (1.59-19.3)**	**< 0.01**	1.02 (0.22-4.6)	0.98	0.54 (0.19-1.56)	0.26	2.94 (0.75-11.5)	0.12	0.22
*E. hirae*	Pan-susceptible	1.33 (0.50-3.55)	0.57	0.67 (0.20-2.21)	0.51	0.64 (0.21-1.99)	0.44	1.27 (0.34-4.74)	0.73	0.06
*E. hirae*	> 1 Antimicrobial Class	0.75 (0.38-1.48)	0.41	1.09 (0.54-2.22)	0.81	1.31 (0.63-2.75)	0.47	0.90 (0.42-1.96)	0.79	0.10
*E. hirae*	Multiclass Resistant	0.79 (0.20-3.21)	0.75	1.14 (0.28-4.67)	0.86	0.99 (0.20-4.85)	0.99	0.69 (0.14-3.44)	0.65	0.28
*E. casseliflavus*	Ciprofloxacin	**4.36 (1.23-15.4)**	**0.02**	0.77 (0.18-3.37)	0.73	1.13 (0.57-2.24)	0.73	**6.39 (2.23-18.3)**	**0.001**	0.07
*E. casseliflavus*	> 1 Antimicrobial Class	1.71 (0.73-3.98)	0.22	0.65 (0.26-1.60)	0.35	1.31 (0.70-2.46)	0.41	**3.45 (1.62-7.34)**	**0.001**	0.17
*E. casseliflavus*	Multiclass resistant	Not Estimable	> 0.99	Not Estimable	> 0.99	3.11 (0.56-17.2)	0.19	7.03 (0.74-67.1)	0.90	0.14

ref-reference group for odds ratio generated by regression analysis.

^a^Intrinsic resistance [33]

When comparing calves in the fall to calves in the spring, *E. casseliflavus* isolates were over four times more likely to be resistant to ciprofloxacin (Table 5). *E. hirae* from calves in the fall were also five times more likely to be resistant to tetracycline than isolates from calves in the spring (Table 5). However, *Enterococcus faecalis* isolates recovered from calves in the spring were nearly 5 times more likely to be resistant to tetracycline compared to isolates from calves in the fall and *E. faecalis isolates* from calves in the spring were less likely to be pan-susceptible than isolates from calves in the fall (Table 5). Resistance to nitrofurantoin was more likely to be seen in isolates from calves in the fall compared to calves in the spring for *E. faecium* (Table 5).

In the spring, calves were more likely to have *E. faecalis* isolates that were resistant tetracycline when compared to cows (Table 5).

Similar to what was seen when comparing calves in the fall to calves in the spring, *E. casseliflavus* isolates from cows in the fall were more likely to be resistant to ciprofloxacin as well as resistant to at least one antimicrobial class than from cows in the spring (Table 5).

### Prevalence of herds with resistance and variability in resistance frequency within herds

The most common resistance detected within herd samples was to lincomycin (range 97 to 100%) (Table 6). The next most common resistance detected within herds samples was tetracycline for isolates from calves in the spring, daptomycin for cows in the spring, ciprofloxacin and daptomycin for calves in the fall, and quinupristin/dalfopristin for cows in the fall (Table 6). Cows in the fall were the only group for which at least one resistant isolate was not observed in every herd (Table 6). The overall prevalence of herds with at least one isolate resistant to a Category I antimicrobial was similar across groups and just slightly higher for isolates from calves in the fall (Table 6).

**Table 6:** Comparison of the proportion of resistance positive herds as well as the average within herd prevalence for resistance positive herds^a^

	Spring Calf (*n* = 39 herds)			Spring Cow (*n* = 39 herds)			Fall Calf (*n* = 39 herds)			Fall Cow (*n* = 36 herds)		
		Within herd prevalence with AMR			Within herd prevalence with AMR			Within herd prevalence with AMR			Within herd prevalence with AMR	
Antimicrobial	Number (%) of herds with AMR	Mean	Min, Max	Number (%) of herds with AMR	Mean	Min, Max	Number (%) of herds with AMR	Mean	Min, Max	Number (%) of herds with AMR	Mean	Min, Max
**Category I: Very High Importance**^b^												
Ciprofloxacin	56% (22)	19%	10, 44%	41% (16)	13%	10, 30%	72% (28)	18%	5, 60%	64% (23)	23%	10, 70%
Daptomycin	72% (28)	29%	10, 50%	62% (24)	23%	10, 56%	72% (28)	18%	6, 50%	58% (21)	21%	10, 60%
Linezolid	5.1% (2)	11%	10, 11%	5.1% (2)	10%	10, 10%	0% (0)	0%	0, 0%	0% (0)	0%	0, 0%
Tigecycline	15% (6)	15%	10, 22%	7.7% (3)	17%	10, 20%	10% (4)	15%	10, 30%	11% (4)	15%	10, 30%
Vancomycin	0% (0)	0%	0, 0%	0% (0)	0%	0, 0%	0% (0)	0%	0, 0%	0% (0)	0%	0, 0%
**Category II: High Importance**^b^												
Erythromycin	26% (10)	16%	10, 50%	18% (7)	16%	10, 40%	26% (10)	10%	5, 20%	11% (4)	10%	10, 10%
Gentamicin	0% (0)	0%	0, 0%	0% (0)	0%	0, 0%	0% (0)	0%	0, 0%	0% (0)	0%	0, 0%
Kanamycin	15% (6)	16%	11, 40%	7.7% (3)	10%	10, 10%	7.7% (3)	10%	10, 10%	0% (0)	0%	0, 0%
Lincomycin	100% (39)	61%	25, 100%	100% (39)	82%	11, 100%	100% (39)	69%	10, 100%	97% (35)	76%	20, 100%
Quinupristin/ dalfopristin	49% (19)	25%	10, 42%	49% (19)	39%	10, 80%	56% (22)	24%	10, 60%	69% (25)	24%	10, 80%
Streptomycin	26% (10)	16%	10, 40%	13% (5)	10%	10, 10%	15% (6)	14%	10, 20%	2.6% (1)	11%	11, 11%
Tylosin	31% (12)	15%	10, 50%	21% (8)	16%	10, 40%	23% (9)	13%	6, 30%	8.3% (3)	13%	10, 20%
**Category III: Medium Importance**^b^												
Chloramphenicol	7.8% (3)	10%	10, 11%	5.1% (2)	10%	10, 10%	10% (4)	9%	6, 10%	0% (0)	0%	0, 0%
Nitrofurantoin	21% (8)	14%	10, 22%	13% (5)	16%	10, 30%	44% (17)	14%	6, 40%	22% (8)	24%	10, 80%
Tetracycline	64% (25)	23%	10, 67%	33% (13)	12%	10, 20%	59% (23)	15%	5, 60%	36% (13)	20%	10, 56%
Pan-susceptible	79% (31)	24%	10, 67%	54% (21)	19%	10, 50%	67% (26)	26%	10, 80%	61% (22)	25%	10, 100%
Herds with ≥ 1 resistant isolate	87% (34)	35%	10, 70%	77% (30)	28%	10, 60%	92% (36)	33%	10, 80%	92% (33)	31%	10, 90%
Multiclass resistant	38% (15)	17%	10, 50%	41% (12)	12%	10, 20%	36% (14)	12%	5, 20%	44% (16)	15%	10, 33%
Category I resistance	82% (32)	42%	11, 100%	79% (31)	25%	10, 75%	87% (34)	32%	11, 80%	83% (30)	33%	10, 89%

^a^Resistance of *E. faecalis* to quinupristin/dalfopristin and lincomycin are not reported because of intrinsic resistance [14]

^b^Government of Canada 2009 [27]

## Discussion

This is the first publication to describe AMR in specific species of *Enterococcus* isolates from western Canadian cow-calf herds and the differences in the relative frequency of these species and associated AMR between cows and calves and between seasons. Three studies from the United States described AMR in enterococci recovered from cow-calf herds in 2007-08 [8] and 2017 [10], and most recently 2019/2020 [20]. More North American data surrounding enteric *Enterococcus* is available from the feedlot sector [1, 7, 12]. Some *Enterococcus* data are also available for retail beef and beef products [1, 19, 35], with one study also examining resistance in *Enterococcus* at the slaughter facility [19].

Slightly higher recover rates were seen in the current publication compared to most of the existing literature [7, 8, 19–21]. The present study followed the same protocol as the new Canadian national surveillance program for both culture on selective media and identification of *Enterococcus* species [9]. Recovery rates of *Enterococcus* in cow-calf focused literature ranged from 48 to 80% in cows [8, 20] and 83% in calves [20], with the exception being the most recent cow-calf study done by the United States Department of Agriculture (USDA) where isolation rates in composite cow and calf samples averaged 98% [10]. One of the two studies with lower recovery rates used a different specific broth and agar for isolation of *Enterococcus* species [8], while the second study used a different commercial selective agar with organism identification based on color change [20]. The most prominent species recovered in the current cow-calf herds was *E. casseliflavus* which was also the primary species recovered in the USDA 2007-08 cow-calf study [8] and the second most recovered species in the 2017 cow-calf samples [1, 10]. Both the present study and the 2017 USDA study used MALDI-TOF MS for species identification, while the 2007-08 USDA study used multiplex PCR [8, 10]. Despite the present study targeting selection of *E. hirae*, *E. hirae* was instead the second most common species isolated, except for calves in the spring, where it was the third most isolated species. *Enterococcus hirae* has also been the predominant isolate recovered in several other studies in beef cattle [7, 10–12, 18, 21], and was the second most prominent isolate in the 2007-08 USDA cow-calf study [1, 8].

While the present study was not designed to describe the species of *Enterococcus* bacteria in cow-calf herds, it did aim to describe AMR in species of interest to human health and when these were not identified, the most common species identified. Selection of isolates for testing varied between studies complicating comparisons to the current work. For example, the most recent study from California of fecal samples from cows and calves required two identifiable isolates per sample and were not speciated [20]. In a recent Alberta feedlot study, 6 isolates were collected per sample with species identity confirmed using sequencing, with 25% of all recovered isolates tested for AMR [1]. Because of limited resources only one isolate was tested per sample in the current study. The 2017 USDA study similarly speciated and saved one isolate per sample for AST, although it wasn’t clear if the isolate was chosen at random or based on a prioritization protocol [10].

The investigation of AMR for *Enterococcus* spp. is further complicated by the extent of intrinsic resistance to some important classes of antimicrobials and variation in intrinsic resistance among species [13]. Intrinsic resistance provides natural resistance to a specific antimicrobial regardless of exposure history and can be due to a lack of affinity of the drug for the bacterial target, the inability of the drug to access bacterial cells, or the presence of drug degrading enzymes [36]. This differs from acquired resistance, which occurs when bacteria obtain the ability to resist the activity of a microbial agent that was previously effective [36].

Common examples of intrinsic resistance for many species of enterococci bacteria include lincomycin and quinupristin/dalfopristin resistance, which are not typically due to exposure and are considered intrinsic [13, 29, 30, 36]. Lincosamide antimicrobials include lincomycin and clindamycin and act by inhibiting protein synthesis [13]. The lnu(B) gene cloned from *E. faecium* and transferred to other enterococcal species is responsible for most of the intrinsic resistance seen in *Enterococcus* [13]. In *E. faecalis,* a *lsa* (lincosamide and streptogramin A resistant gene) provides all *E. faecalis* with intrinsic resistant to lincomycin and streptogramins such as quinupristin/dalfopristin [14].

Species-specific differences in resistance to daptomycin has been observed for enterococci bacteria and the breakpoints for determining resistance are not considered accurate for all species [28]. Daptomycin MIC values differ for *E. faecalis* and *E. faecium*, with a greater proportion *E. faecium* having increased MIC values [28]. For *E. faecalis* and *E. faecium* resistance levels are typically low but increasing resistance has been observed via both acquired and intrinsic resistance mechanisms [29, 30]. One study credits the development of daptomycin resistance in both species to alterations in the phospholipid and fatty acid composition of the cell membrane [29], while another has found genetic differences between the resistant and non-resistant bacteria [30].

Regardless of the source, daptomycin resistance is a concern, because the drug is used to treat vancomycin-resistant bacteria [29], a common problem in human medicine [15]. Concerns around the breakpoints used for daptomycin, specifically the variation in species MIC values, make a standard comparison of all enterococci values difficult [28]. Thus, the prevalence of daptomycin resistance, particularly for *E. hirae*, observed in this study should be interpreted with caution particularly as reports of daptomycin resistance in *Enterococcus* species isolates from cattle are limited [10, 19, 21, 37]. Recent studies have either not included it in their testing protocols [1, 12, 20] or have not provided reports for individual species or species other than *E. faecium* and *E. faecalis* [10, 19, 37]. However, Messele et al. did report similar daptomycin AST results for *E. hirae* to those observed in the present study using the same AST protocol and that resistance to daptomycin in *E. hirae* increased from feedlot entry to exit [21].

Like the Canadian processing and retail beef study, the USDA study focusing on cow-calf herds, and the Australian study lincomycin was the most common resistance in the present study. Resistance to lincomycin ranged from 48 to 100% in American cow-calf herds [8, 10], 61% in calves leaving Australian cow-calf operations [21] and 94-100% in Canadian beef study [19]. Lincomycin resistance prevalence was not available in the feedlot study from Alberta, but lincosamide resistant genes were found in isolates [1].

Quinupristin/dalfopristin, a member of the streptogramins class, was also a common resistance target, with resistance observed across all animal classes, seasons, and *Enterococcus* species. Sixteen percent of all isolates and 14% of isolates from calves in the fall were resistant to quinupristin/dalfopristin lower than that from pooled samples from American cow-calf herds (26%) [10]. The data from calves near the time of weaning in the fall is of interest as this is the group most closely related to feedlot cattle in the production cycle. In Alberta feedlots, resistance to quinupristin/dalfopristin was much lower than observed in this study, averaging 3% [1].

Quinupristin/dalfopristin is approved for the treatment of antimicrobial resistant Gram-positive bacteria, specifically for the treatment of vancomycin resistant enterococci [38]. Resistance to quinupristin/dalfopristin in food animals has been linked to the use of virginiamycin as a feed additive, an antimicrobial similar in structure and mechanism [38]. In Canada, the use of virginiamycin is approved for use in cattle being fed for slaughter to help reduce liver abscesses [39]. However, virginiamycin use has not been reported in cow-calf herds [17]. A single sampled herd had an associated feedlot allowing for potential limited exposure [17]; however, no virginiamycin use was reported in any of the sampled herds in 2020. Resistance to quinupristin/dalfopristin varied between herds with herd of origin having a slight impact on resistance for *E. casseliflavus*.

The CLSI and the European Committee on Antimicrobial Susceptibility Testing (EUCAST), a division of the European Society of Clinical Microbiology and Infectious Diseases, listed expected resistance phenotypes based on intrinsic resistance patterns observed in bacterial species. *E. casseliflavus* was described as intrinsically resistant to quinupristin/dalfopristin by in both reports [33, 34]. The high prevalence of *E. casseliflavus* isolates resistant to quinupristin/dalfopristin in the current study is more likely a result of intrinsic resistance than as a result of exposure to a structural analogue such as virginiamycin.

Resistance to the fluoroquinolones class of antimicrobials was also relatively common and was identified in most of the multiclass resistance combinations. This antimicrobial class includes the category I antimicrobial ciprofloxacin. Resistance to this antimicrobial, considered very important to human health averaged 12% in calves, and 10% in cows. This is lower than what was seen in the United States in 2017, where resistance to ciprofloxacin for all tested species averaged 16% [10]. Ciprofloxacin resistance was most common in *E. faecium* in both the current publication and the American study [10]. In Canadian herds in 2020, the use of fluoroquinolones at least once was low with only 4.8% (*n* = 7) herds reporting the use of fluoroquinolones at least once in the most commonly treated classes of nursing calves and cows [17]. However, none of the herds in the present study reported use of fluoroquinolones during 2020 [17]. Use data were not available for all sampled herds in 2021.

One of the most common antimicrobials used on cow-calf operations is tetracycline [8, 40]. Overall, *E. faecalis* resistant isolates from cows in the current study averaged 8.5% similar to that in the most recent American cow-calf study [20]. In western Canada in 2014, 84% of herds from the Canadian Cow-Calf Surveillance Network (C3SN) reported using oxytetracycline on their operation at least once [40]. Resistance to tetracycline varied from 2.6 to 11% for *E. faecalis*, and between 18 and 38% for other *Enterococcus* species in American cow-calf herds [8, 10]. In one feedlot study, resistant of *E. faecalis* isolates to tetracycline was 38% [1], nearly triple the current prevalence in fall calves (15%). Further, 65% *E. hirae* isolates were resistant to tetracycline [7] in another feedlot study where tetracyclines can be used in feed for prevention of histophilosis or for injectable metaphylaxis [16, 41]. Resistance to tetracycline via *tetL* and *tetM* determinants were linked to the mobile plasmid pM7M2 [2].

While macrolides were used at least once by 56% of Canadian cow-calf herds [17], less than 3% of *Enterococcus* isolates were resistant to macrolides in the current study. Frequency of macrolide use within herds was low, with approximately half (48%) of herds reporting the use of macrolides treating less than 5% of their herd [17]. Resistance to macrolides in Canadian feedlots was greater than in the current publication, with 83% of *E. hirae* isolates resistant to tylosin, and 72% of *E. hirae* isolates resistant to erythromycin [7, 9]. Elevated resistance in feedlots is potentially associated with injectable and in-feed macrolide use for metaphylaxis and liver abscess control [16] where use is reported in most feedlots and at least once in most animals, particularly those at high risk for bovine respiratory disease. This differs from the pattern of reported use in cow-calf herds where the highest use was reported in calves before weaning, but less than 4% of herds reported use in more than 30% of animals [17]. Unlike the USDA study, which found one isolate to be vancomycin-resistant [8], no vancomycin resistance was seen in the current publication, or other western Canadian publications [1, 9]. The CDC estimated that vancomycin resistant enterococci (VRE) caused nearly 55,000 infections and 5400 deaths in 2017 [15]. In Canada, VRE infections tripled in hospitals between 2007 and 2013, with VRE infections associated with *E. faecium* [42]. Differences in the profiles of *Enterococcus* species as well as resistance profiles between bovine feces and human isolates suggest that *Enterococcus* from beef cattle is unlikely to be an important factor in *Enterococcus* infections in the human population, specifically for VRE [1].

Multiclass resistance in the current publication was lower than resistance to at least three classes of antimicrobials in Australian calves at feedlot arrival [21]. Overall, 9.6% of isolated recovered from calves at feedlot induction were resistant to at least three antimicrobial classes, compared to 4.4% in the current publication [21]. Differences in multiclass resistance levels between the current study and other previous publications is in part due to the differences in defining multiclass resistance. For example, the Australian feedlot study notes the issue of intrinsic resistance in evaluating multiclass resistance but does not specify whether intrinsic resistance was included or excluded from the calculation [21] as was done in the present study per recommendations from Sweeny et al. (2018) [43]. The current study and CIPARS defines multiclass resistance as resistance to three or more classes of antimicrobials [44]; whereas, another western Canadian publication defined multiclass resistance as resistance to at least two classes of antimicrobials [1]. In Alberta feedlots, 37 and 33% of *E. hirae* isolates recovered from bovine feces and feedlot catch-basins were resistant to two or more antimicrobial classes, respectively [1]. In the current study, 28% of all *Enterococcus* isolates were resistant to two or more antimicrobial classes, whereas 51% of all *E. hirae* isolates were resistant to two or more classes of antimicrobials.

As was previously report by Messele et al. [21], the relatively high frequency of multiclass resistance is not unexpected given the capacity for horizontal gene transfer by species within the *Enterococcus* genus. But as is noted in [21] and is more challenging to explain in these multiclass resistance patterns is the varying prevalence of resistances to drugs and classes of drug not used in veterinary medicine where reported intrinsic resistance does not explain the findings as well as resistance to drugs that are occasionally used but where use was not reported in these herds. Examples of interest include daptomycin resistance in *E. hirae*; ciprofloxacin resistance in *E. casseliflavus*, ciprofloxacin, quinupristin/dalfopristin, and nitrofurantoin resistance in *E. faecium*; and non-sensitivity to tigecycline in *E. faecium*. Further whole genome sequencing work is needed to understand the mechanisms of horizontal resistance transfer in this population.

While the comparison to other literature from across North America and Australia allows for a more in-depth understanding of the AMR profiles and prevalence observed in the current publication, methodological differences did limit direct comparisons. Antimicrobial susceptibility testing using disk diffusion methodology was reported in multiple studies [1, 7, 12]. The differences in methodology resulted in variation between other *Enterococcus* species studies and the current publication, specifically in the antimicrobials being tested, including the antimicrobials for which some of the highest resistance was seen in the current publication such as ciprofloxacin, lincomycin, and quinupristin/dalfopristin.

Broth microdilution used for susceptibility testing used in the current publication was also reported in other studies examining *Enterococcus* resistance in cow-calf herds and retail beef [19–21, 35], including at the national surveillance level in Canada [9]. The same Sensititre plate used in the current publication was also used in other studies, allowing for the most direct comparison of findings [9, 19, 21]. In the one recent cow-calf study from California, a different Sensititre plate was used limiting the potential for direct comparison to other data [20]. The BOPO7F plate contains a different panel of antimicrobials, specifically including antimicrobials approved for the treatment of bovine respiratory disease (BRD) and subsequent reporting susceptibility for three of the tested antimicrobials for *Enterococcus* species, resulting in resistance to at least one antimicrobial in only 15% of isolates [20].

Questions about differences in AMR prevalence for *Enterococcus* species between spring and fall and cows and calves were based on findings from similar studies of *E. coli*. Previous work on Canadian beef calves found that *E. coli* from calves in the spring was nearly 10 times more likely to be resistant to at least one antimicrobial when compared to isolates from calves in the fall [23]. Similarly, *E. coli* isolates recovered from calves in the spring were 10 times and 7.1 times more likely to be resistant to at least one antimicrobial than cows from the same herd in samples collected in 2002 and 2003 respectively [24]. More recent data from 2021, also identified higher prevalence of resistance to sulfisoxazole, tetracyclines, and chloramphenicol in calves in the spring compared to calves in the fall or cows in the spring [45]. The current study found that resistance also varied by animal class as well as season. However, that variance was species and antimicrobial dependent. A potential factor in contributing to differences in resistance levels between calves in the spring and calves in the fall is physiological differences between calves at an early age in the spring versus a few months of age at weaning [23, 46]. Calves had a decrease in the number of resistant *E. coli* relative to susceptible *E. coli* as they aged [46].

Antimicrobial use and the AMR status of the herd have been shown to be a predictor of AMR profiles in calves [47]. Antimicrobial use is highest in cow-calf herds in the spring and the highest numbers of calves are treated for respiratory disease and diarrhea [16, 17, 40]. Antimicrobial use practices in Californian cow-calf herds were shown to account for approximately a 20% variation on AMR data [20]. Resistance of *Enterococcus* to macrolides was linked to antimicrobial exposure history in feedlot cattle. Groups of feedlot cattle receiving treatment with one of three macrolides (tilmicosin, tulathromycin, or tylosin) were shown to be 76 times more likely to have erythromycin resistance in the first 28 days post-treatment compared to cattle that received no antimicrobials, and 66 times more likely to have erythromycin resistance compared to pre-treatment [11].

In the present study, the observed differences between cows and calves and between the spring and the fall varied in whether they might reasonably be explained by antimicrobial use. *E. faecalis* isolates from calves were more likely to be resistant to tetracycline in the spring as compared to calves in the fall and to cows in the spring or fall. However, the exact opposite observation was made for *E. hirae*, where isolates from calves in the fall had significantly higher tetracycline resistance. While 53% of herds reported any tetracycline use in nursing calves in a 2020 study [17], only 12% of herds reported use in more than 5% of animals. The differences in numbers do not explain the reported difference patterns for either weaned calves with 39% of herds reporting use and 10% reporting use in more than 5% of calves and for cows with 71% of herds reporting use and 13% reporting use in more than 5% of cows. Similarly, statistically significant spring to fall differences in resistance to ciprofloxacin, which was not used in these herds, and nitrofurantoin, which is not approved for veterinary use, could not be explained by AMU.

While the current study was successful in providing baseline data regarding *Enterococcus* in cow-calf herds from western Canada, there are limitations to the findings. Firstly, the sample size of the current study was relatively small, initially recruiting 50 herds with 10 samples per animal class per herd per season. However, only 39 herds provided samples in both the spring and fall sampling period, reducing study power for comparison, and potentially reducing the generalizability of the results. Producers reported environmental conditions limited participation. The summer of 2021 was extremely dry across western Canada, forcing some producers to reduce herd size and for others increasing the resources and time needed to secure pasture, feed stores and water supplies for their herds.

Secondly, cow-calf pairs were not deliberately sampled, nor were the same animals deliberately sampled in the spring and fall to reduce the burden of sample collection. The resulting sampling scheme made it impossible to measure effects related to exposure via the dam or changes in individual animals from one sample period to the next. Third, only a single isolate from each of the sampled animals was analysed which might not fully represent the AMR profile of an animal. Finally, because participating herds were part of a volunteer surveillance network, the sample population likely represents relatively progressive, intensively managed operations but does allow for direct comparison to most other research and surveillance reports. Despite the limitations, the distribution of herds did reflect the expected frequency of cow-calf herds in western Canada. The proportion of herds from each province was very similar to the relative distribution of beef cows within western Canada based on the 2021 Agriculture Census: Alberta 51 vs 49%, Saskatchewan 23 vs 33%, Manitoba 13 vs 12%, and British Columbia 13 vs 5% [48].

While the AMR research in the cow-calf sector is growing, additional surveillance and genomics studies are required to better understand the AMR profiles seen for *Enterococcus* species, such as *E. casseliflavus,* that were most commonly recovered from cow-calf herds. This baseline information is critical to assess the impact of antimicrobial stewardship initiatives. For example, recent policy changes such as those seen in December 2018 requiring all veterinary antimicrobials to be placed under a prescription only status [49]. Antimicrobial use practices on these western Canadian cow-calf herds are published elsewhere [8, 17, 40]. The potential relationships between AMU and AMR will be the subject of future analyses.

## Conclusion

While the study was successful in describing the prevalence of AMR in *Enterococcus* obtained from western Canadian cow-calf herds, resistance in *Enterococcus* is complicated due to limitations in current understanding of intrinsic resistance and the accuracy of breakpoints. The results must be interpreted with caution. Future research will be required to better understand the accuracy of resistance profiles and how representative observed AMR profiles are of *Enterococcus* in the cow-calf environment.

## Methods

### Producer recruitment

Producers were recruited from participants in the Canadian Cow-Calf Surveillance Network (C3SN). The C3SN included producers from all regions across Canada with 56 participants from its predecessor, the Western Canadian Cow-Calf Surveillance Network (WCCCSN) [40].

Herds were recruited for the C3SN through consultation with veterinarians, advertisements through research agencies such as the Beef Cattle Research Council (BCRC), provincial beef organizations, and word of mouth. Recruitment targeted herds larger than 40 breeding animals who reported pregnancy checking and had basic calving and production records [50]. Additionally, access to email was requested to allow for efficient communication.

Initial recruitment for the fecal sampling project occurred using a survey released to C3SN participants in June of 2020. Producers were eligible for fecal sampling if they were from the western Canadian provinces of British Columbia, Alberta, Saskatchewan, or Manitoba and answered “yes” to be willing to share treatment records. Eligible producers were contacted in December of 2020 to evaluate their interest in participating in the fecal AMR project.

Fifty herds initially agreed to participate in the fecal AMR project: 25 (50%) from Alberta, 12 (24%) from Saskatchewan, 8 (16%) from Manitoba, and 5 (10%) from British Columbia. Thirty-nine herds provided fecal samples in both the spring and fall. Of those herds, 20 (51%) were from Alberta, nine (23%) were from Saskatchewan, five (13%) herds were from British Columbia and five (13%) herds were from Manitoba.

Fecal samples were collected on-farm by the herd veterinarian using a sampling kit and instructions supplied by the study team. Samples were collected from 10 randomly selected cows and 10 calves per operation in the spring and the fall. Producers were not required to target cow-calf pairs or to sample the same animals in the spring and fall. Feces were collected fresh, either directly from the rectum of an animal or from fresh fecal pats immediately following deposition using individual gloves and placed in sterile screw top containers. Fecal samples were shipped in a cooler with ice packs to the Western College of Veterinary Medicine, Saskatoon, SK. Samples were then catalogued and submitted to the regional diagnostic laboratory (Prairie Diagnostic Services Inc. (PDS), Saskatoon, SK) for bacterial culture and antimicrobial susceptibility testing.

### Laboratory methods

The fecal samples were weighed, and 4.0 g was transferred into 50 ml centrifuge tubes containing 1% buffered peptone water. The mixture was vortexed thoroughly and placed in a mixer for 1 hour. The pre-enrichment mixtures were incubated at 35C for 18-24 hrs under ambient atmospheric conditions. One ml of the incubated pre-enrichment mixture was transferred to 1 ml of Brucella broth containing 15% glycerol and frozen at -80C for future use. The remaining fecal sample was saved in a plastic container and frozen at -80C.

A selective medium for *Enterococcus* spp., mEnterococcus agar (Oxoid, Fisher Scientific, Waltham, Massachusetts, USA) was inoculated with 10 μl of the pre-enrichment mixture. The plates were streaked to obtain isolated colonies. The mEnterococcus agar plates were incubated at 35C for 48 hours under 5% CO2 conditions.

After incubation, plates were examined for the presence of typical *Enterococcus* spp. colonies. Up to six colonies exhibiting different morphological characteristics (color – pink or red, size – tiny, small, or large) per plate were sub-cultured on Columbia agar with 5% sheep blood to obtain pure colonies. The sub-cultured plates were incubated at 35C for 18-24 hours under 5% CO2 conditions. The colonies grown on blood agar were identified using Matrix-Assisted Laser Desorption Ionization Time-Of-Flight mass spectrometry (MALDI-TOF MS) according to the manufacturer guidelines. The MALDI-TOF MS Biotyper Microflex LT (Bruker Daltonik, Bremen, Germany) Compass version 1.4 software and MSP library were used for direct testing.. An internal calibration control before each sample identification used a standard extract of *E. coli* DH5 alpha to confirm the characteristic peptide and protein profile in MALDI-TOF MS. Positive, negative, and blank controls were also processed for each day of sample set up and for each new media lot using *Enterococcus faecalis* ATCC 29212 and *E. coli* ATCC 25922. Only scores > 2 and indicating secure species-level identification were used for further analysis. Pure cultures from the target organisms used for antimicrobial susceptibility testing (AST) by broth microdilution were saved in Tryptic Soy Broth containing 15% glycerol in duplicates and stored at -80C.

Minimum inhibitory concentrations (MICs) were measured according to Clinical and Laboratory Standards Institute (CLSI) guidelines [32, 33] using NARMS CMV3AGPF Sensititre plates recommended for determining MICs for Gram-positive bacteria (Thermo Fisher Scientific, Waltham, Massachusetts, USA). The NARMS CMV3AGPF Sensititre plates [51] included doubling dilutions of 16 antimicrobials across the specified concentrations ranges (ug/ml): chloramphenicol (2-32), ciprofloxacin (0.12-4), daptomycin(0.25-16), erythromycin (0.25-8), gentamicin (128-1024), kanamycin (128-1024), lincomycin (1-8), linezolid (0.5-8), nitrofurantoin (2-64), penicillin (0.25-16), quinupristin/dalfopristin (0.5-32), streptomycin (512-2048), tetracycline(1-32), tigecycline (0.015-0.5), tylosin (0.25-32) and vancomycin (8-32). One *Enterococcus* spp. isolate was selected from each sample for AST prioritizing *E. faecalis* first, then *E. faecium*, because both are important to human health [1, 7, 29]. *E. hirae*, the most common species identified in feedlot cattle [1, 7] was selected last if present. If none of the above specified *Enterococcus* spp. were identified, AST was performed for the most common *Enterococcus* spp. isolated from that sample.

As a commercial test kit was used for susceptibility testing, the manufacturer’s instructions and recommendations were followed for quality control (QC) testing [33]. Briefly, the 0.5 McFarland turbidity equivalent bacterial broth was prepared from pure bacterial isolate using Sensititre Nephelometer (ThermoFisher Scientific, Nepean, ON, Canada). The dosing broths were prepared by transferring 10 μl of the suspension to 11 ml of a Sensititre Cation Adjusted AutoRead Muller-Hinton Broth w/TES (ThermoFisher Scientific, Nepean, ON, Canada). The dosing broth was inoculated onto Sensititre plate using Sensititre AIM Automated Inoculation System (ThermoFisher Scientific, Nepean, ON, Canada). The inoculated plates were tightly sealed using non-perforated adhesive seals and incubated at 35C for 18-24 hours under ambient atmospheric conditions. MICs were interpreted as susceptible (S), intermediate (I), or resistant (R) based on CLSI human breakpoints using a BioMic V3 system (Giles Scientific Inc., Santa Barbara, California, USA), and a manual mirror box confirmation if necessary to ensure growth was present. The MIC is determined by evaluating the panel for the first well without visible growth.

The control strain used in the laboratory was *E. faecalis* ATCC 29212. Control strain stock cultures were maintained at a minimum of -20 °C. Subcultured isolates were maintained between 2 °C and 8 °C. Quality control testing occurred as per NARMS laboratory methods which included weekly testing and whenever a new lot of sterile water, broth, or panel was used [52]. Additionally, for each new panel lot number uninoculated broth was dispensed into the plate and incubated to test sterility. All results were within accepted quality control ranges [33].

### Data management and analysis

Data were managed using commercial database and spreadsheet programs (Microsoft Access and Excel, Microsoft, Redmond Washington, USA). Primary outcomes of interest were presence or absence of specific species of *Enterococcus* and then whether the organism was resistant to each antimicrobial for which there were human CLSI guidelines for interpretation for the *Enterococcus* species [32]. Resistance was reported at the isolate level, with isolates having intermediate MIC values being classified as susceptible. Multiclass resistance was defined by an isolate being resistant to ≥3 classes of antimicrobials [44]. Pan-susceptibility included isolates that were susceptible to all classes of antimicrobials.

Reports of *E. faecalis* resistance to either lincomycin or quinupristin/dalfopristin were considered to be intrinsic resistance and were not included in any data summaries or risk factor analysis [14, 33]. There was further uncertainty regarding meaningful AST breakpoints, potentially associated with intrinsic resistance, for lincomycin [13, 14] and daptomycin [28–30]. Based on recommendations in Sweeney et al. 2018 [43], all intrinsic resistance identified in the CLSI documentation [33] was excluded from the summary of multiclass resistance [33].

Random effects logistic regression was used to measure differences in the relative frequency of recovered isolates for each species of interest as well as the occurrence of resistance for isolates of each species between cows and calves within each season and then between seasons for both cows and calves. When the prevalence of resistance where resistance was not classified as intrinsic, occurrence of multiclass resistance, or occurrence of pan-susceptibility were > 5%, data were examined for statistical differences in resistance to specific antimicrobials. All models were constructed using a commercial software program (STATA version 16.1, StataCorp, College Station, Texas, USA). Logistic regression models included a random effect for herd, and fixed effects for season, cow vs calf and the interaction between animal type and season. Odds ratios (OR) described the relative differences in outcomes among groups and *P* < 0.05 was considered statistically significant. In addition to determination of odds ratios, intracluster correlation coefficients (ICCs) were reported. ICCs were used as a measure of clustering of outcomes within herd after accounting for differences between animal classes and seasons.

### Supplementary Information

**Additional file 1.**

## Data Availability

Not applicable.
