# Supplementary material for: Antimicrobial resistance in Enterococcus isolated from western Canadian cow-calf herds
Source: BMC Vet Res. 2024 Jan 3;20:6. doi: 10.1186/s12917-023-03843-6 (PMC10763084; doi:10.1186/s12917-023-03843-6)
Supplement: Supplementary file 1 — Additional file 1. [file 12917_2023_3843_MOESM1_ESM.docx]

**Supplementary Materials:**

**Antimicrobial resistance in *Enterococcus* isolated from western Canadian cow-calf herds**

Jayce D. Fossen^1^, John R. Campbell^1^, Sheryl P. Gow^2^, Nathan Erickson^1^, Cheryl L. Waldner^1^

^1^Large Animal Clinical Sciences, Western College of Veterinary Medicine, 52 Campus Dr, Saskatoon, Saskatchewan, S7N 5B4

^2^Public Health Agency of Canada, Saskatoon, Saskatchewan, S7N 5B4

Corresponding author: Cheryl Waldner, cheryl.waldner@usask.ca

**Appendix A: Descriptive summary of MIC results**

**Table A1:** Minimum Inhibitory Concentration (MIC) range for selected *Enterococcus* isolates recovered in the spring of 2021 from 390 calves and 387 cows from 39 herds.

| Relative frequency of isolates | Enterococcus isolates from calves (n=349) in the spring | | | | | Enterococcus isolates from cows (n=385) in the spring | | | | |
| --- | --- | --- | --- | --- | --- | --- | --- | --- | --- | --- |
|  | *E. faecalis* | *E. casseliflavus* | *E. hirae* | *E. faecium* | *E. species ^A^* | *E. casseliflavus* | *E. hirae* | *E. faecalis* | *E. faecium* | *E. species ^B^* |
|  | 24%  (85/349) | 23%  (79/349) | 21%  (75/349) | 14% (50/349) | 17%  (60/349) | 42% (162/385) | 18%  (70/385) | 8.8% (34/385) | 7.0% (27/358) | 24%  (92/385) |
| Antimicrobials tested: | MIC Range (µg/mL) | | | | | | | | | |
|  | **Category I: Very High Importance ^#^** | | | | | | | | | |
| Ciprofloxacin | 0.5-4 | 0.5->4 | 0.5-4 | 1->4 | 0.12-4 | 0.25->4 | 0.25-4 | 0.5-2 | 2->4 | 0.25-4 |
| Daptomycin * | 0.5-16 | 0.25-8 | 1-16 | 0.5-16 | 0.5-16 | 0.25-4 | 1-16 | 1-4 | 2-16 | 0.25-16 |
| Linezolid | 1-8 | 1-4 | 1-4 | 0.5-4 | 0.5-4 | 0.5-4 | 1-4 | 2-8 | 1-4 | 0.5-8 |
| Tigecycline ** | 0.03-0.5 | 0.03-0.25 | 0.015-0.5 | 0.015-0.5 | 0.015-0.5 | 0.03-0.5 | 0.03-0.25 | 0.03-0.25 | 0.03-0.5 | 0.03-0.25 |
| Vancomycin *** | 0.5-8 | 0.5-8 | *0.25-8* | 0.25-8 | 0.5-8 | *0.5-8* | 0.5-8 | 1-4 | 0.25-2 | 0.5-8 |
|  | **Category II: High Importance ^#^** | | | | | | | | | |
| Erythromycin | 0.25->8 | 0.25-8 | 0.25-8 | 0.25-8 | 0.25-2 | 0.25-8 | 0.25-8 | 0.25->8 | 0.25-4 | 0.25-8 |
| Gentamicin *** | 128 | *128* | *128* | *128* | 128 | *128* | 128 | *128* | *128* | 128 |
| Kanamycin *** | 125->1024 | *128* | *128-1024* | *128-1024* | 128 | *128-1024* | 128-256 | *128* | *128-1024* | 128-512 |
| Lincomycin *** | *NA* | *8->8* | *1->8* | *1->8* | *1->8* | *1->8* | 1->8 | *NA* | 1->8 | 1->8 |
| Penicillin | 0.5-8 | 0.25-4 | 0.25-8 | 0.25-8 | 0.25-4 | 0.25-8 | 0.25-8 | 2-4 | 0.25-16 | 0.25-16 |
| Quinupristin/ dalfopristin *** | *NA* | *2-8* | *0.5-8* | 0.5-16 | 0.5-4 | *0.5-16* | 0.5-4 | *NA* | 0.5-4 | 0.5-32 |
| Streptomycin *** | 512->2048 | *512-1024* | *512-2048* | *512-2048* | 512 | *512-2048* | 512 | *512-1024* | *512* | 512-1024 |
| Tylosin | 2-32 | 1->32 | 1->32 | 1-32 | 1-32 | 1->32 | 1->32 | 2-32 | 1-8 | 0.25-32 |
|  | **Category III: Medium Importance ^#^** | | | | | | | | | |
| Chloramphenicol | 0.25-32 | 4-16 | 4-8 | 4-8 | 2-8 | 2-16 | 4-8 | 4-32 | 2-8 | 2-8 |
| Nitrofurantoin | 8-64 | 8-16 | 8-64 | 8->64 | 4->64 | 2-64 | 8-64 | 16 | 32->64 | 2->64 |
| Tetracycline | 1->32 | 1->32 | 1->32 | 1->32 | 1->32 | 1->32 | 1->32 | 1->32 | 1->32 | 1->32 |

**A, B:** *E. durans, E. gallinarum, E. mundtii, E. species;* ^#^ Government of Canada 2009 [27]* Daptomycin breakpoints for susceptible vary for *E. faecium* as compared to other species [28–31]; **** No CLSI breakpoint for resistance for tigecycline, rather only for susceptible; the numbers reflect isolates that are not susceptible vs resistant. [32]; *** *Intrinsic resistance*: aminoglycosides (gentamicin, kanamycin, streptomycin), (*E.* spp) [33], lincosamides, (*E. faecalis*) [14, 33, 34], (*E. casseliflavus*) [34]; quinupristin/dalfopristin (*E. faecalis*) [14, 33, 34], (*E. casseliflavus*) [33, 34]; vancomycin (*E. casseliflavus*) [33];  *NA – E . faecalis resistance to either lincomycin or quinupristin/dalfopristin were not included in any analysis due to intrinsic resistance due to the presence of the lsa gene* [14].

**Table A2:** Minimum Inhibitory Concentration (MIC) range for selected *Enterococcus* isolates recovered in the fall of 2021 from 419 calves from 39 herds and 359 cows from 36 herds.

| Relative frequency of isolates | Enterococcus isolates from calves (n=413) in the fall | | | | | Enterococcus isolates from cows (n=358) in the fall | | | | |
| --- | --- | --- | --- | --- | --- | --- | --- | --- | --- | --- |
|  | *E. casseliflavus* | *E. hirae* | *E. faecalis* | *E. faecium* | *E. species ^A^* | *E. casseliflavus* | *E. hirae* | *E. faecalis* | *E. faecium* | *E. species ^B^* |
|  | 36% (149/413) | 22%  (89/413) | 15%  (62/413) | 11%  (45/413) | 16% (68/413) | 37% (131/358) | 16%  (56/358) | 13%  (48/358) | 12%  (43/358) | 22%  (80/358) |
| Antimicrobials tested: | MIC Range (µg/mL) | | | | | | | | | |
|  | **Category I: Very High Importance ^#^** | | | | | | | | | |
| Ciprofloxacin | 0.5->4 | 0.5-4 | 0.5-2 | 0.5->4 | 0.25-2 | 1->4 | 0.5-4 | 0.5-4 | 1->4 | 0.12-4 |
| Daptomycin * | 0.25-4 | 1-16 | 0.5-16 | 1-8 | 0.25-16 | 0.25-8 | 0.5-16 | 1-8 | 2-8 | 0.25-16 |
| Linezolid | 1-4 | 1-4 | 1-2 | 1-4 | 1-4 | 1-4 | 1-4 | 1-2 | 0.5-4 | 1-4 |
| Tigecycline ** | 0.03-0.5 | 0.03-0.25 | 0.06-0.25 | 0.03-0.5 | 0.03-0.5 | 0.03-0.5 | 0.03-0.5 | 0.06-0.25 | 0.015-0.5 | 0.03-0.5 |
| Vancomycin *** | *0.5-8* | 0.5-8 | 1-8 | 0.5-8 | 0.5-8 | *2-8* | 0.5-8 | 0.5-8 | 0.5-8 | 0.25-8 |
|  | **Category II: High Importance ^#^** | | | | | | | | | |
| Erythromycin | 0.25-8 | 0.25->8 | 0.25->8 | 0.25-8 | 0.25-2 | 0.25-8 | 0.25-4 | 0.25-4 | 0.25-8 | 0.25-4 |
| Gentamicin *** | *128* | 128 | *128* | *128* | 128 | *128* | 128-256 | *128* | *128* | 128 |
| Kanamycin *** | *125-256* | 128->1024 | *128->1024* | *128-256* | 128 | *128-256* | 128 | *128* | *128-256* | 128-512 |
| Lincomycin *** | *1->8* | *1->8* | NA | *1->8* | *1->8* | *4->8* | 1->8 | *NA* | 1->8 | 1->8 |
| Penicillin | 0.25-8 | 0.25-8 | 0.5-4 | 0.25-8 | 0.25-2 | 0.25-2 | 0.25-8 | 0.25-4 | 0.25-8 | 0.25-8 |
| Quinupristin/ dalfopristin *** | *0.5-8* | 0.5-4 | *NA* | 0.5-4 | 0.5-4 | *0.5-4* | 0.5-4 | *NA* | 0.5-8 | 0.5-8 |
| Streptomycin *** | *512* | 512-1024 | *512->2048* | *512* | 512 | *512* | 512-1024 | *512* | *512* | 512 |
| Tylosin | 1->32 | 1->32 | 2->32 | 2-8 | 1->32 | 1->32 | 1-4 | 2-4 | 2-8 | 0.5-4 |
|  | **Category III: Medium Importance ^#^** | | | | | | | | | |
| Chloramphenicol | 4-32 | 2-16 | 8->32 | 4-8 | 2-8 | 4-16 | 4-8 | 4-8 | 2-8 | 2-8 |
| Nitrofurantoin | 8-64 | 2-64 | 8-32 | 16->64 | 8->64 | 2-64 | 8->64 | 8-64 | 32->64 | 2->64 |
| Tetracycline | 1 | 1->32 | 1->32 | 1->32 | 1->32 | 1->32 | 1->32 | 1->32 | 1->32 | 1->32 |

**A, B:** *E. durans, E. gallinarum, E. mundtii, E. species;* ^#^ Government of Canada 2009 [27]* Daptomycin breakpoints for susceptible vary for *E. faecium* as compared to other species [28–31] **** No CLSI breakpoint for resistance for tigecycline, rather only for susceptible; the numbers reflect isolates that are not susceptible vs resistant. [32]; *** *Intrinsic resistance*: aminoglycosides (gentamicin, kanamycin, streptomycin), (*E.* spp) [33], lincosamides, (*E. faecalis*) [14, 34], (*E. casseliflavus*) [34]; quinupristin/dalfopristin (*E. faecalis*) [14, 33, 34], (*E. casseliflavus*)[33, 34]; vancomycin (*E. casseliflavus*) [33];  *NA – E . faecalis resistance to either lincomycin or quinupristin/dalfopristin were not included in any analysis due to intrinsic resistance due to the presence of the lsa gene* [14].

**Table A3:** Minimum Inhibitory Concentrations (MICs) summarized as MIC50 values (medians) for selected *Enterococcus* isolates recovered in the spring of 2021 from 390 calves and 387 cows from 39 herds.

| Relative frequency of isolates | Enterococcus isolates from calves (n=349) in the spring | | | | | Enterococcus isolates from cows (n=385) in the spring | | | | |
| --- | --- | --- | --- | --- | --- | --- | --- | --- | --- | --- |
|  | *E. faecalis* | *E. casseliflavus* | *E. hirae* | *E. faecium* | *E. species ^A^* | *E. casseliflavus* | *E. hirae* | *E. faecalis* | *E. faecium* | *E. species ^B^* |
|  | 24%  (85/349) | 23%  (79/349) | 21%  (75/349) | 14% (50/349) | 17%  (60/349) | 37% (131/358) | 16%  (56/358) | 13%  (48/358) | 12%  (43/358) | 22%  (80/358) |
| Antimicrobials tested: | MIC50 (µg/mL) | | | | | | | | | |
|  | **Category I: Very High Importance ^#^** | | | | | | | | | |
| Ciprofloxacin | 1 | 2 | 0.5 | 4 | 1 | 2 | 0.5 | 1 | 4 | 0.5 |
| Daptomycin * | 2 | 1 | 8 | 4 | 2 | 1 | 8 | 2 | 4 | 2 |
| Linezolid | 2 | 2 | 2 | 2 | 2 | 2 | 2 | 2 | 2 | 2 |
| Tigecycline ** | 0.12 | 0.12 | 0.06 | 0.12 | 0.12 | 0.12 | 0.09 | 0.12 | 0.12 | 0.12 |
| Vancomycin *** | 1 | *4* | 1 | 0.5 | 0.5 | *4* | 1 | 1 | 0.5 | 0.5 |
|  | **Category II: High Importance ^#^** | | | | | | | | | |
| Erythromycin | 1 | 1 | 0.25 | 1.5 | 0.5 | 1 | 0.25 | 0.5 | 2 | 0.5 |
| Gentamicin *** | *128* | *128* | 128 | *128* | 128 | *128* | 128 | *128* | *128* | 128 |
| Kanamycin *** | *128* | *128* | 128 | *128* | 128 | *128* | 128 | *128* | *128* | 128 |
| Lincomycin *** | *NA* | *8* | 8 | 8 | 8 | *8* | 8 | *NA* | 1 | 8 |
| Penicillin | 4 | 0.5 | 2 | 2 | 1 | 0.5 | 2 | 4 | 4 | 1 |
| Quinupristin/ dalfopristin *** | *NA* | *2* | 2 | 1.5 | 2 | *2* | 2 | *NA* | 0.5 | 2 |
| Streptomycin *** | *512* | *512* | 512 | *512* | 512 | *512* | 512 | *512* | *512* | 512 |
| Tylosin | 2 | 2 | 2 | 4 | 2 | 2 | 2 | 2 | 4 | 2 |
|  | **Category III: Medium Importance ^#^** | | | | | | | | | |
| Chloramphenicol | 8 | 8 | 4 | 8 | 8 | 8 | 4 | 8 | 8 | 8 |
| Nitrofurantoin | 16 | 8 | 32 | 64 | 32 | 8 | 32 | 16 | 64 | 32 |
| Tetracycline | 1 | 1 | 1 | 1 | 1 | 1 | 1 | 1 | 1 | 1 |

**A, B:** *E. durans, E. gallinarum, E. mundtii, E. species;* ^#^ Government of Canada 2009 [27]* Daptomycin breakpoints for susceptible vary for *E. faecium* as compared to other species [28–31] **** No CLSI breakpoint for resistance for tigecycline, rather only for susceptible; the numbers reflect isolates that are not susceptible vs resistant. [32]; *** *Intrinsic resistance*: aminoglycosides (gentamicin, kanamycin, streptomycin), (*E.* spp) [33], lincosamides, (*E. faecalis*) [14, 34], (*E. casseliflavus*) [34]; quinupristin/dalfopristin (*E. faecalis*) [14, 33, 34], (*E. casseliflavus*)[33, 34]; vancomycin (*E. casseliflavus*) [33];  *NA – E . faecalis resistance to either lincomycin or quinupristin/dalfopristin were not included in any analysis due to intrinsic resistance due to the presence of the lsa gene* [14].

**Table A4:** Minimum Inhibitory Concentrations (MICs) summarized as MIC50 values (medians) for selected *Enterococcus* isolates recovered in the fall of 2021 from 419 calves from 39 herds and 359 cows from 36 herds.

| Relative frequency of isolates | Enterococcus isolates from calves (n=413) in the fall | | | | | Enterococcus isolates from cows (n=358) in the fall | | | | |
| --- | --- | --- | --- | --- | --- | --- | --- | --- | --- | --- |
|  | *E. casseliflavus* | *E. hirae* | *E. faecalis* | *E. faecium* | *E. species ^A^* | *E. casseliflavus* | *E. hirae* | *E. faecalis* | *E. faecium* | *E. species ^B^* |
|  | 36% (149/413) | 22%  (89/413) | 15%  (62/413) | 11%  (45/413) | 16% (68/413) | 37% (131/358) | 16%  (56/358) | 13%  (48/358) | 12%  (43/358) | 22%  (80/358) |
| Antimicrobials tested: | MIC50 (µg/mL) | | | | | | | | | |
|  | **Category I: Very High Importance ^#^** | | | | | | | | | |
| Ciprofloxacin | 2 | 0.5 | 2 | 4 | 1 | 2 | 0.75 | 2 | 3 | 1 |
| Daptomycin * | 2 | 4 | 2 | 4 | 2 | 2 | 8 | 2 | 4 | 2 |
| Linezolid | 2 | 2 | 2 | 2 | 2 | 2 | 2 | 2 | 2 | 2 |
| Tigecycline ** | 0.12 | 0.12 | 0.12 | 0.12 | 0.12 | 0.12 | 0.12 | 0.12 | 0.12 | 0.12 |
| Vancomycin *** | *4* | 1 | 1 | 0.5 | 0.5 | *4* | 1 | 1 | 0.5 | 0.5 |
|  | **Category II: High Importance ^#^** | | | | | | | | | |
| Erythromycin | 2 | 0.25 | 1 | 2 | 0.25 | 2 | 0.25 | 1 | 2 | 0.5 |
| Gentamicin *** | *128* | 128 | *128* | *128* | 128 | *128* | 128 | *128* | *128* | 128 |
| Kanamycin *** | *128* | 128 | *128* | *128* | 128 | *128* | 128 | *128* | *128* | 128 |
| *Lincomycin **** | *8* | *8* | *NA* | *1* | *8* | *8* | *8* | *NA* | *8* | *8* |
| Penicillin | 0.5 | 2 | 4 | 4 | 1 | 0.5 | 2 | 4 | 4 | 1 |
| Quinupristin/ dalfopristin *** | *2* | 2 | NA | 0.5 | 2 | *2* | 2 | NA | 2 | 2 |
| Streptomycin *** | *512* | 512 | *512* | *512* | 512 | *512* | 512 | *512* | *512* | 512 |
| Tylosin | 2 | 2 | 2 | 4 | 2 | 2 | 2 | 2 | 4 | 3 |
|  | **Category III: Medium Importance ^#^** | | | | | | | | | |
| Chloramphenicol | 8 | 8 | 8 | 8 | 8 | 8 | 8 | 8 | 8 | 7 |
| Nitrofurantoin | 8 | 32 | 16 | 64 | 32 | 8 | 32 | 16 | 64 | 32 |
| Tetracycline | 1 | 1 | 1 | 1 | 1 | 1 | 1 | 1 | 1 | 1 |

**A, B:** *E. durans, E. gallinarum, E. mundtii, E. species;* ^#^ Government of Canada 2009 [27]* Daptomycin breakpoints for susceptible vary for *E. faecium* as compared to other species [28–31] **** No CLSI breakpoint for resistance for tigecycline, rather only for susceptible; the numbers reflect isolates that are not susceptible vs resistant. [32]; *** *Intrinsic resistance*: aminoglycosides (gentamicin, kanamycin, streptomycin), (*E.* spp) [33], lincosamides, (*E. faecalis*) [14, 34], (*E. casseliflavus*) [34]; quinupristin/dalfopristin (*E. faecalis*) [14, 33, 34], (*E. casseliflavus*)[33, 34]; vancomycin (*E. casseliflavus*) [33];  *NA – E . faecalis resistance to either lincomycin or quinupristin/dalfopristin were not included in any analysis due to intrinsic resistance due to the presence of the lsa gene* [14].

**Appendix B: Results for *Enterococcus* summarized across all species**

**Table B1:** Prevalence of antimicrobial resistance (%) summarized for all *Enterococcus* isolates recovered in 2021 with population-averaged 95%CI accounting for clustering by herd.

|  | **Cows and Calves in 2021^NA^** |
| --- | --- |
|  | n=1505 isolates, 39 herds |
| **Category I: Very High Importance ^#^** |  |
| Ciprofloxacin | 10.7% (8.6-12.7%) |
| Daptomycin * | 13.7% (11.0-16.5%) |
| Linezolid | 0.3% (0.0-0.6%) |
| Tigecycline ** | 1.7% (0.8-2.5%) |
| Vancomycin *** | 0.0% (0.0-%) |
| **Category II: High Importance ^#^** |  |
| Erythromycin | 2.7% (1.6-3.8%) |
| Gentamicin *** | 0.0% (0.0-%) |
| Kanamycin *** | 1.0% (0.3-1.7%) |
| Lincomycin *** | 72% (68-77%) |
| Penicillin | 0.1% (0.0-0.3%) |
| Quinupristin/ dalfopristin *** | 15.2% (11.6-19.0%) |
| Streptomycin *** | 2.0% (1.1-2.9%) |
| Tylosin | 3.1% (1.7-4.5%) |
| **Category III: Medium Importance ^#^** |  |
| Chloramphenicol | 0.6% (0.2-1.0%) |
| Nitrofurantoin | 4.1% (2.5-5.6%) |
| Tetracycline | 8.6% (6.5-10.7%) |

^#^ Government of Canada 2009 [27]* Daptomycin breakpoints for susceptible vary for *E. faecium* as compared to other species [28–31]; **** No CLSI breakpoint for resistance for tigecycline, rather only for susceptible; the numbers reflect isolates that are not susceptible vs resistant. [32]; *** *Intrinsic resistance*: aminoglycosides (gentamicin, kanamycin, streptomycin), (*E.* spp) [33], lincosamides, (*E. faecalis*) [14, 34], (*E. casseliflavus*) [34]; quinupristin/dalfopristin (*E. faecalis*) [14, 33, 34], (*E. casseliflavus*) [33, 34]; vancomycin (*E. casseliflavus*) [33]; *NA – E . faecalis resistance to either lincomycin or quinupristin/dalfopristin were not included in any analysis due to intrinsic resistance due to the presence of the lsa gene* [14].

**Table B2:** Prevalence of antimicrobial resistance (%) summarized for all *Enterococcus* isolates recovered in the spring and fall of 2021 with 95% confidence intervals accounting for clustering by herd.

|  | **Antimicrobial resistance Enterococcus (isolates from all species^NA^)** | | | |
| --- | --- | --- | --- | --- |
|  | **Spring** | | **Fall** | |
|  | **Calves** | **Cows** | **Calves** | **Cows** |
|  | n=349, 39 herds | n=385, 39 herds | n=413, 39 herds | n=358, 36 herds |
|  | **Category I: Very High Importance #** | | | |
| **Ciprofloxacin** | 10.7% (7.0-14.4%) ^a^ | 5.2% (2.9-7.5%) ^b^ | 12.3% (8.3-16.3%) ^a^ | 14.7% (9.1-20.3%) ^a^ |
| **Daptomycin *** | 21.5% (15.6-27.4%) ^a^ | 8.5% (4.1-13.0%) ^b^ | 13.5% (9.4-17.5%) ^b^ | 12.2% (7.6-16.9%) ^b^ |
| **Linezolid** | 0.6% (0.0-1.4%) | 0.5% (0.0-1.2%) | 0.0% (0.0-0.9%) | 0.0% (0.0-1.0%) |
| **Tigecycline **** | 2.3% (0.5-4.2%) | 1.3% (0.0-2.8%) | 1.4% (0.0-3.1%) | 1.6% (0.0-3.5%) |
| **Vancomycin ***** | 0.0% (0.0-1.1%) | 0.0% (0.0-1.0%) | 0.0% (0.0-0.9%) | 0.0% (0.0-1.0%) |
|  | **Category II: High Importance #** | | | |
| **Erythromycin** | 4.3% (1.2-7.5%) ^a^ | 2.8% (0.4-5.2%) | 2.6% (1.2-4.1%) | 1.1% (0.0-2.1%) ^b^ |
| **Gentamicin** *** | 0.0% (0.0-1.1%) | 0.0% (0.0-1.0%) | 0.0% (0.0-0.9%) | 0.0% (0.0-1.0%) |
| **Kanamycin** *** | 2.6% (0.2-5.1%) ^a^ | 0.8% (0.0-1.6%) | 0.8% (0.0-1.6%) ^b^ | 0.0% (0.0-1.0%) |
| **Lincomycin ***** | 62% (56-68%) ^a^ | 82% (76-88%) ^b^ | 69% (61-77%) ^a,c^ | 75% (66-83%) ^b,c^ |
| **Penicillin** | 0.0% (0.0-1.1%) | 0.5% (0.0-1.2%) | 0.0% (0.0-0.9%) | 0.0% (0.0-1.0%) |
| **Quinupristin/ dalfopristin ***** | 12.7% (7.9-17.5%) | 18.7% (10.8-26.6%) | 13.3% (8.0-18.7%) | 16.5% (10.7-22.3%) |
| **Streptomycin** *** | 4.3% (1.5-7.1%) ^a^ | 1.3% (0.2-2.4%) | 2.2% (0.5-3.9%) | 0.3% (0.0-0.8%) ^b^ |
| **Tylosin** | 5.2% (2.0-8.3%) ^a^ | 3.4% (0.7-6.0%) | 3.0% (1.0-5.0%) | 1.0% (0.0-2.3%) ^b^ |
|  | **Category III: Medium Importance #** | | | |
| **Chloramphenicol** | 0.9% (0.0-1.8%) | 0.5% (0.0-1.2%) | 1.0% (0.1-1.9%) | 0.0% (0.0-1.0%) |
| **Nitrofurantoin** | 2.9% (0.9-4.8%) | 2.1% (0.2-4.0%) ^a^ | 6.3% (3.4-9.3%) ^b^ | 4.6% (1.2-8.1%) |
| **Tetracycline** | 15.6% (10.0-21.3%) ^a^ | 3.9% (2.0-5.7%) ^b^ | 8.8% (5.3-12.2%) ^c^ | 6.8% (2.8-10.9%) ^b,c^ |

^a,b,c,d^ Different superscripts within row reflect significant differences (p<0.05) between risk groups.
^#^ Government of Canada 2009 [27]* Daptomycin breakpoints for susceptible vary for *E. faecium* as compared to other species [28–31]; **** No CLSI breakpoint for resistance for tigecycline, rather only for susceptible; the numbers reflect isolates that are not susceptible vs resistant. [32]; *** *Intrinsic resistance*: aminoglycosides (gentamicin, kanamycin, streptomycin), (*E.* spp) [33], lincosamides, (*E. faecalis*) [14, 34], (*E. casseliflavus*) [34]; quinupristin/dalfopristin (*E. faecalis*) [14, 33, 34], (*E. casseliflavus*) [33, 34]; vancomycin (*E. casseliflavus*) [33]; *NA – E . faecalis resistance to either lincomycin or quinupristin/dalfopristin were not included in any analysis due to intrinsic resistance due to the presence of the lsa gene* [14].
